# Cavitating Electrohydrodynamic Flow in the Vicinity of a Bio-Inspired Electrode Surface

**DOI:** 10.3390/biomimetics11080557

**Published:** 2026-08-05

**Authors:** Jing Li, Alexander Hernandez, Beatrice Boatemaa, Xuewei Zhang

**Affiliations:** Department of Electrical Engineering and Computer Science, Texas A&M University-Kingsville, Kingsville, TX 78363, USA; jing.li@students.tamuk.edu (J.L.); alexander.hernandez@students.tamuk.edu (A.H.); beatrice.boatemaa@tamuk.edu (B.B.)

**Keywords:** electrohydrodynamic flow, cavitation, electrode, pulsed high voltage

## Abstract

Electrostrictive cavitation is a mechanism of the electrical breakdown of dielectric liquids under nanosecond pulsed high voltages and a promising way of controlled nanoscale cavity generation. To better understand electrostrictive cavitation in water, this work develops a cavitating electrohydrodynamic model to simulate the distribution of tensile stress. Compared with the continuum electrohydrodynamic model, the tensile stress from the new model is reduced wherever cavitation has initiated. Further, an innovative electrode design concept inspired by the cell membrane is proposed, in which the electrode is hollow with a permeable enclosure, allowing liquid flow in response to a pressure difference between the interior and the outside. The simulations based on the cavitating electrohydrodynamic model suggest that this electrode design results in even lower tensile stress near the high-voltage electrode surface and holds potential to suppress cavitation and subsequent electrical breakdown.

## 1. Introduction

The electrical breakdown of liquid dielectrics under nanosecond (ns) or sub-nanosecond (sub-ns) pulsed electric fields has been studied extensively since the early 2010s [1,2,3,4,5]. The research conducted in this relatively young field has made great contributions to the theories of electrical breakdown in liquid dielectrics by introducing a novel mechanism, electrostrictive cavitation, that is uniquely applicable to cases at the ns and sub-ns timescales [6]. Additionally, it is also of fundamental importance to the improved design of plasma sources for various applications in biomedical engineering [7,8] and enhanced electrical insulation for pulsed high-voltage equipment in electric power and other systems [9,10]. For water, in particular, the understanding of the mechanisms and processes of ns/sub-ns electrical breakdown sheds new light on and presents a promising approach to cavitation research [11,12], one of the important subfields of fluid mechanics.

Previous studies [1,2,3,4,5] have focused either on the diagnostics of the breakdown process and discharge plasma, or on the verification of the electrostrictive cavitation prior to breakdown. More recently, there have been more theoretical and computational investigations of the electrostrictive cavitation mechanism [13,14,15,16,17,18]. In water stressed by highly inhomogeneous, ns-pulsed electric fields, electrostriction could result in the formation of a cavitation zone where the total pressure drops below the cavitation threshold of the liquid. The cavitation zone in turn sets the stage for the generation and multiplication of primary electrons that seed the electrical breakdown (streamer discharge). However, a complete picture of the physical mechanisms and processes involved has yet to be constructed. As the intermediate stage between the generation of tensile stress due to electrostriction and the initiation of the first plasma discharge channels, cavitation dynamics demand research attention to gain a detailed knowledge of the cavitation zone, e.g., the density and size of the cavities formed.

On the other hand, although there has been little work done, purposeful modifications of the electrostrictive cavitation process are desired in many applications, from electrical insulation (to prevent breakdown initiation) and plasma sources (to adjust discharge characteristics) to emerging techniques such as nanocavity-based drug delivery [19] and nanocavity-based reactors [20]. The conventional wisdom is to manipulate the applied pulsed voltages or use liquids with prescribed properties. Inspired by the cell membrane, which is a flexible and permeable interface separating the interior from the environment and triggering responses to changes in conditions such as salt concentration [21], this work proposes a new type of electrode that could introduce some ways to modify the process of electrostrictive cavitation. In particular, if the aim is to suppress cavitation development via reducing tensile stress in the vicinity of the electrode surface, one approach is to introduce an outflow from the interior of the electrode (assuming constant pressure) in response to the pressure difference across the permeable electrode surface. To test the feasibility of this concept, new numerical studies are called for.

The objective of this work is to develop a cavitating electrohydrodynamic (EHD) model to investigate the effect of the bio-inspired electrode surface on electrostrictive cavitation. To achieve this, the investigation is divided into three modeling stages. First, the basic EHD model is implemented to examine the pressure response of water stressed by highly inhomogeneous, ns-pulsed electric fields. This model highlights the effect of the electrostrictive force. Second, the model is extended to a cavitating EHD model using the volume of fluid (VOF) method. This stage examines how the introduction of the cavity phase changes the pressure after cavitation inception. Third, the Darcy boundary condition is introduced to represent the proposed high-voltage electrode design. Compared with the previous model, the potential effect of the Darcy boundary on regulating cavitation is evaluated.

The anticipated contributions and significance of the work have two main aspects. First, coupling the continuum EHD model with a cavitating mixture model, this work demonstrates a novel approach to gain insights into the characteristics of the cavitation zone during the initial stages of ns liquid breakdown. In addition, this work proposes and examines an innovative electrode design concept that holds potential to suppress cavitation and subsequent breakdown.

The organization of the rest of the paper is as follows. In Section 2, a detailed description of the physical and numerical models used in this study is provided. In Section 3, representative results are presented for the purpose of the verification and validation of the models and methods; furthermore, the dynamics of pressure-field regulation and cavitation suppression via the bio-inspired electrode are evaluated. In Section 4, the concepts and methods of the study are discussed. In Section 5, the conclusions are drawn.

## 2. Model

**Figure 1:**
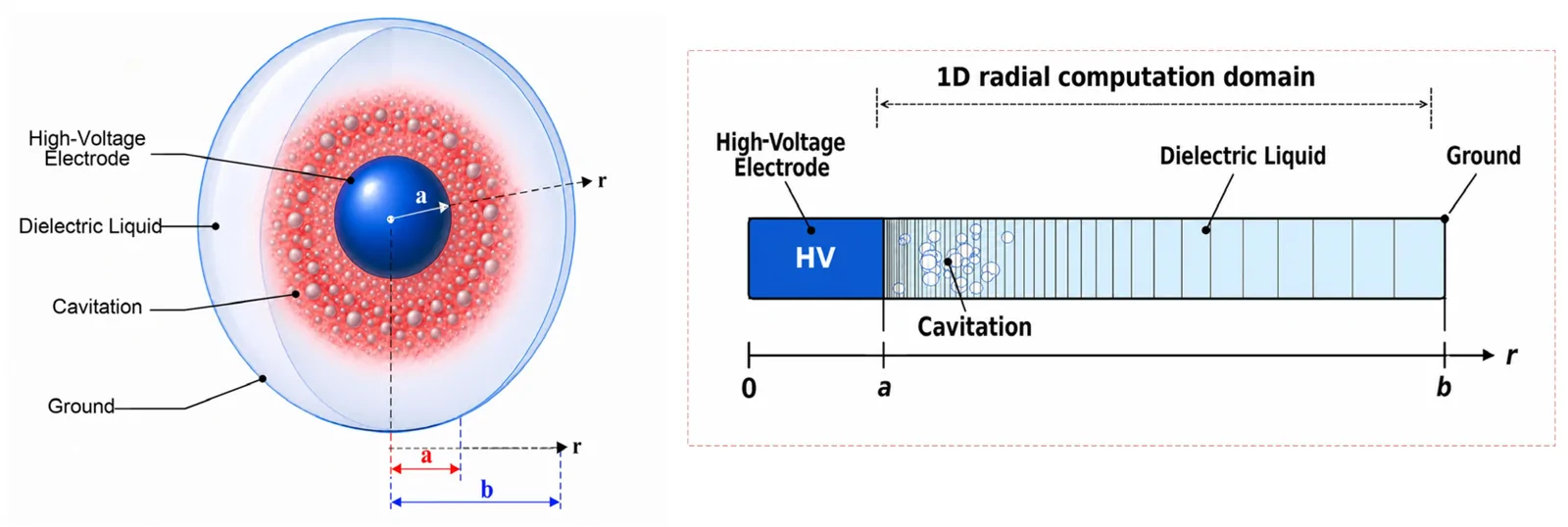
Illustration of the geometrical configuration of the problem (**left**) and the mesh setting for the spherically symmetrical (1D) case (**right**).

As illustrated in Figure 1, this work considers a simple, spherically symmetric system consisting of an inner electrode of radius a=40 μm, a concentric outer electrode of radius b=200 μm, and water filling the region between them. The outer electrode is grounded, while the inner electrode’s electric potential is a linear function of time, i.e., $V\left(t\right)=ct$, where c is a coefficient that represents the rising slopes of the voltage pulse.

This study focuses on the electromechanical response of the dielectric liquid on ns timescales. In the absence of electrical breakdown, the governing equations for the dielectric liquid stressed by an inhomogeneous, ns-pulsed electric field are as follows [3]:

(1) $$\frac{\partial \rho_{m}}{\partial t}+\nabla \cdot \left(\rho_{m}u\right)=0$$

(2) $$\rho_{m}\left(\frac{\partial u}{\partial t}+u\cdot \nabla u\right)=-\nabla p+F$$

(3) $$p=\left(p_{0}+B\right){\left(\frac{\rho_{l}}{\rho_{0}}\right)}^{\gamma }-B$$

where $\rho_{m}$ is the mass density of the liquid–cavity mixture, u is the velocity field, p is the hydrodynamic pressure, and F is the volume force density due to the applied electric field. Equation (3) is called the Tait equation of state, with B, $\rho_{0}$, and γ as constants and $\rho_{l}$ denoting the density of liquid water.

Neglecting free charge effects and dielectric inhomogeneity, the volumetric force density is reduced to the electrostrictive term:

(4) $$F=\frac{\varepsilon_{0}}{2}\nabla \left(E^{2}\frac{\partial \varepsilon }{\partial \rho }\rho \right)$$

where $\varepsilon_{0}$ is the vacuum permittivity, ε is the relative permittivity of the dielectric liquid, and E is the electric field magnitude. Under these conditions, the electric field between the two concentric spherical electrodes is:

(5) $$E\left(r,t\right)=\frac{V\left(t\right)}{r^{2}\left(\frac{1}{a}-\frac{1}{b}\right)}\hat{r}$$

where $\hat{r}$ is the unit vector in the radial direction and r is the radial coordinate. For water, an approximate relation $\frac{\partial \varepsilon }{\partial \rho }\rho \approx \beta \varepsilon$ can be applied to Equation (4), yielding the simplified form $F\approx \frac{\beta \varepsilon \varepsilon_{0}}{2}\nabla E^{2}=-\nabla p_{E}$, where $p_{E}=\frac{-\beta \varepsilon \varepsilon_{0}}{2}E^{2}$ is the tensile stress (negative pressure) due to electrostriction. When the electric field is strong enough and highly divergent, the corresponding force F (proportional to the gradient of the square of the electric field magnitude) drives the liquid, initially at rest, into motion.

Solving the above EHD model, previous research has shown evidence of the total pressure $p_{total}=p+p_{E}$ dropping below $p_{th}$, the critical pressure for cavitation (in this study, $p_{th}=-30$ MPa is used [22]), which indicates the initiation of cavitation. However, the above EHD model is only valid for the liquid as a continuum; once cavities are present in the liquid, it will be necessary to use a cavitating EHD model that describes the mixture of liquid and cavities.

A straightforward approach is to adopt the VOF method for multiphase flows [23]. The mixture density is the weighted average of the densities of liquid and vapor (cavity) phases:

(6) $$\rho_{m}=\alpha \rho_{l}+\left(1-\alpha \right)\rho_{v}$$

where $\rho_{v}$ is the vapor density in cavities and α is the liquid volume fraction defined in each computational cell (α=1 for all liquid, α=0 for all vapor). In the present case, the liquid fraction remains close to unity throughout the domain. The mixture density is reasonably approximated as $\rho_{m}\approx \alpha \rho_{l}$.

The transport of the liquid volume fraction is governed by

(7) $$\frac{\partial \alpha }{\partial t}+\nabla \cdot \left(\alpha u\right)=\frac{\dot{m}}{\rho_{l}}$$

where $\dot{m}={\dot{m}}_{1}+{\dot{m}}_{2}$ is the net mass transfer rate due to condensation (${\dot{m}}_{1}$) and vaporization/cavitation (${\dot{m}}_{2}$), obtained from the Schnerr–Sauer finite mass transfer (FMT) model [24,25]:

(8a) $${\dot{m}}_{1}=C_{1}\alpha \left(1-\alpha \right)\frac{3\rho_{l}\rho_{v}}{\rho R_{B}}\sqrt{\frac{2}{3\rho_{l}\left|p-p_{th}\right|}}max\left(p-p_{th},0\right)$$

(8b) $${\dot{m}}_{2}=C_{2}\alpha \left(1+\alpha_{Nuc}-\alpha \right)\frac{3\rho_{l}\rho_{v}}{\rho R_{B}}\sqrt{\frac{2}{3\rho_{l}\left|p-p_{th}\right|}}min\left(p-p_{th},0\right)$$

where $R_{B}=\sqrt[{3}]{\frac{3}{4\pi n_{0}}\frac{1+\alpha_{Nuc}\;-\;\alpha }{\alpha }}$ is the representative cavity radius, and $\alpha_{Nuc}=\frac{\frac{\pi n_{0}d_{Nuc}^{2}}{6}}{1\;+\;\frac{\pi n_{0}d_{Nuc}^{2}}{6}}$ is the equilibrium nucleus volume fraction. The parameters $n_{0}$ and $d_{Nuc}$ denote the nucleus number density per unit volume and the characteristic nucleation site diameter. In [24,25], the context of which is boiling, the value of $d_{Nuc}$ is in the order of 10 μm. However, in the case of electrostrictive cavitation, the cavities cannot grow to this size at the nanosecond timescale [18]. The nuclei are the empty space between molecules; therefore, the diameter and density should be in the same order of molecule size (10^−10^ m) and density (10^28^ m^−3^). Consequently, one has $\alpha_{Nuc}\ll 1$ and $R_{B}$ ~ 10 nm. If varying the values of ($n_{0}$, $d_{Nuc}$) to make $\alpha_{Nuc}$ change in the range of 10^−2^ to 10^−6^, the value of $R_{B}$ would remain in the order of 10 nm and the change in the model results would also be insignificant. The constants $C_{1}$ and $C_{2}$ are the condensation and vaporization coefficients used in OpenFOAM-based implementations [26].

The cavitating EHD model, Equations (1)–(8), will be numerically solved in one-dimensional spherical symmetry, i.e., all physical quantities varying only with the radial coordinate r. The initial conditions are $p\left(t=0\right)=p_{0}=10^{5}$ Pa and u(t=0)=0. The following default boundary conditions are applied in the radial domain r∈[a, b]:

(9) $${\left.\frac{\partial \rho }{\partial r}\right|}_{r=a}=0,\;\;{\left.\frac{\partial \rho }{\partial r}\right|}_{r=b}=0,\;\;{u|}_{r=a}=0,\;\;{\left.\frac{\partial u}{\partial r}\right|}_{r=b}=0\;$$

where r=a corresponds to the inner electrode surface and r=b denotes the outer boundary of the computational domain. These conditions represent a no-slip constraint at the electrode surface, symmetry at the axis, and zero-gradient (Neumann) conditions for density and velocity at the outer boundary.

In the proposed design concept, the high-voltage electrode is hollow, with its interior filled with the same liquid at pressure $p_{0}$. The bio-inspired electrode surface is modeled by imposing the Darcy boundary condition at r=a, which allows controlled outflow across the permeable surface according to Darcy’s law:

(10) $${u|}_{r=a}=-\frac{k}{\mu L}\left(p_{total}\left(a,t\right)-p_{0}\right)$$

where k is the permeability of the thin, porous layer (the electrode surface), μ is the dynamic viscosity of the liquid, and L is the effective thickness of the porous layer.

The governing equations are solved using a one-dimensional finite-volume formulation in spherical coordinates. Mass and momentum equations are written in conservative form and discretized using cell-centered control volumes with fluxes evaluated at cell faces, ensuring the conservation of mass and momentum. Spatial gradients are approximated using second-order centered reconstructions, while the volume-fraction equation is advanced using a VOF transport scheme with a finite mass-transfer source term for cavitation. Time integration is performed explicitly using a forward-Euler method with adaptive time stepping based on CFL and source-term stability constraints. At each time step, the pressure, permittivity, and electric field are updated consistently, and electrostrictive forces are included in the momentum balance. Cavitation inception is detected using the total pressure. When the local total pressure falls below a prescribed threshold, the phase-change source term is activated.

## 3. Results

**Figure 2:**
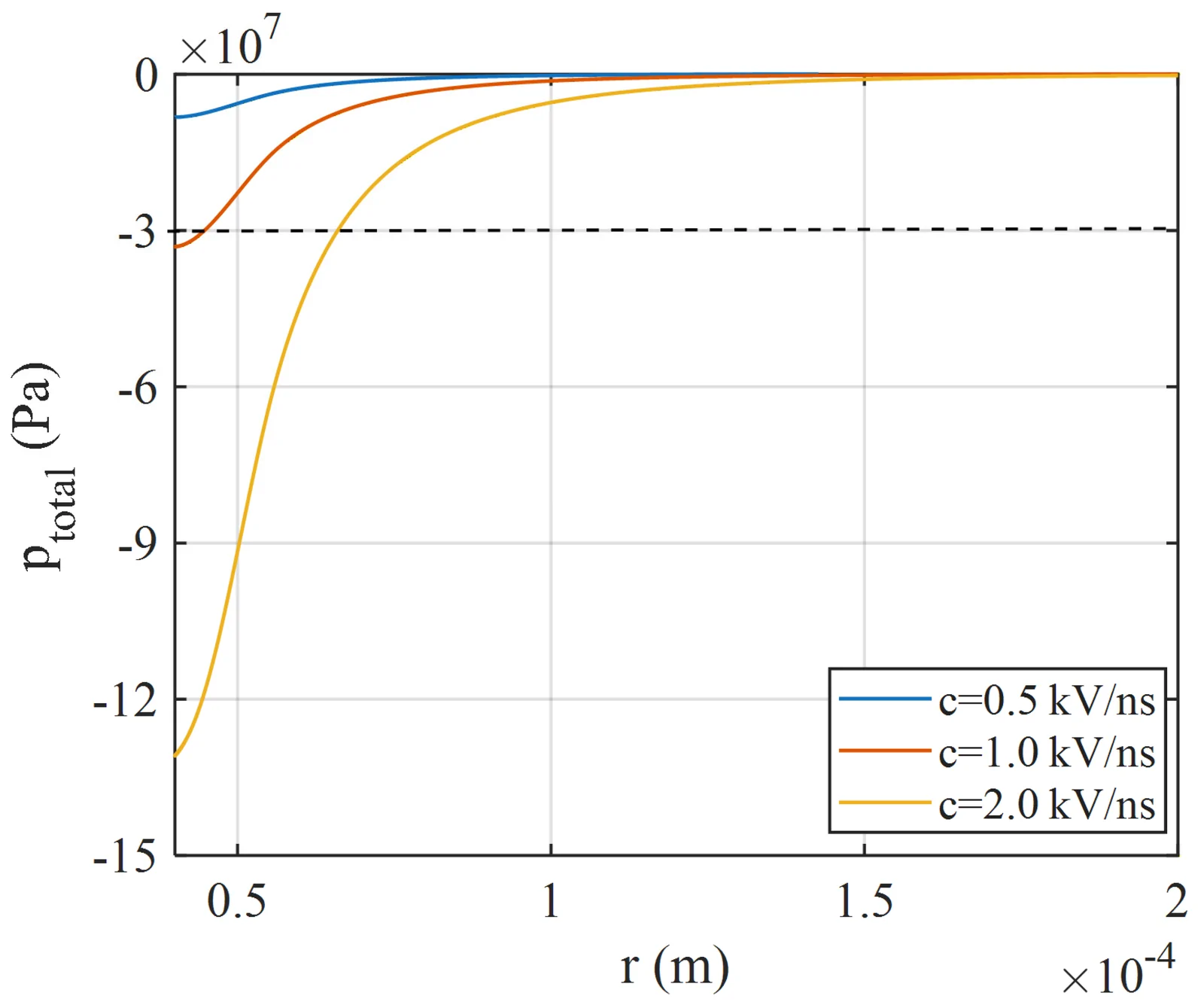
Total pressure profiles (t=10 ns) for different voltage levels using the EHD model. The voltages start at 0 kV and increase linearly, i.e., $V\left(t\right)=ct$, where c= 0.5, 1, and 2 kV/ns. The dashed line indicates the critical pressure for cavitation, $p_{th}=-30$ MPa.

**Figure 3:**
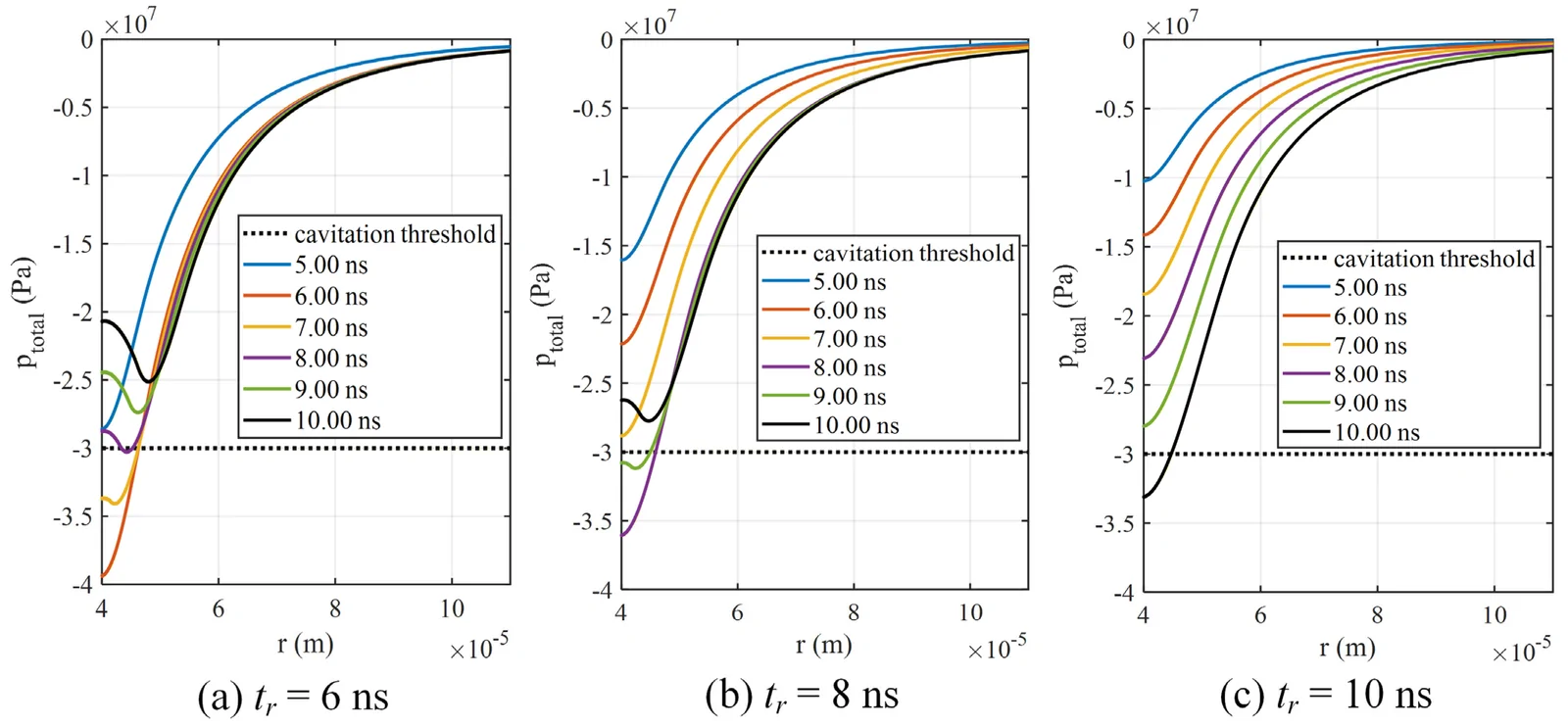
Total pressure profiles for different voltage rise times ($t_{r}$) using the EHD model. The rise times are set to 6 ns (**a**), 8 ns (**b**), and 10 ns (**c**). The voltages start from 0 V, increase linearly to 10 kV at $t_{r}$, and maintain the same voltage level afterwards. To highlight the differences among the pressure profiles in the vicinity of the inner electrode surface, only part of the inter-electrode gap (from r=40 μm to 110 μm) is shown.

As the baseline, the EHD model (without the VOF or Darcy boundary condition) is first solved. The results are presented in Figure 2 and Figure 3. Figure 2 shows the radial distribution of total pressure ($p_{total}$) at t=10 ns at three different voltage levels. As the slope of the voltage is increased from 0.5 kV/ns to 2 kV/ns, the total pressure near the inner electrode (r=40 μm) drops to increasingly lower levels. This is attributed to the electrostrictive tensile stress $p_{E}=\frac{-\beta \varepsilon \varepsilon_{0}}{2}E^{2}$, which is proportional to $E^{2}$. When the maximum voltage reaches 10 kV, the total negative pressure just exceeds the cavitation threshold. In reported experiments [4,5], the high-voltage electrode is a needle and the radius of curvature of the needle tip is between 30 μm and 50 μm; the Schlieren/shadowgraph technique indicates that the minimum voltage required to create a detectable cavitation zone is between 9 kV and 13 kV. The basic EHD model results in Figure 2 are in good agreement with the experimental observations.

Figure 3 shows the progression over time of the radial distribution of total pressure with three different voltage rise times. Comparing the three cases, it can be observed that:

(i) Before the voltages reach the peak (10 kV), the negative pressure is strongest at the surface of the inner electrode and attenuates toward the outer electrode. As the voltage is increasing steeply, the electrostriction is dominant while the fluid has been set into motion to substantially change the pressure distribution.
(ii) With the same peak voltage, a shorter rise time results in more negative total pressure at the inner electrode surface. In the extreme case, the voltage is increasing so slowly that fluid flow can be fully developed to relax the electrostrictive tensile stress. This is why the phenomena occur at the ns timescale.
(iii) Once the voltage plateaus, the negative pressure “wave” propagates away from the inner electrode while the tensile stress is significantly relaxed due to fluid motion. These features have also been reported in previous computational studies [2,3]. During pressure relaxation, the voltage is constant at the peak value; the electrostrictive forcing still exists and drives fluid flow toward the inner electrode surface. With more and more fluid incoming, the pressure at the inner electrode surface will be raised more than the pressure farther away from the electrode surface. In the region even farther away from the electrode surface (the right half of the U-shaped curves), the pressure is still dominated by electrostriction and little changes are made.

**Figure 4:**
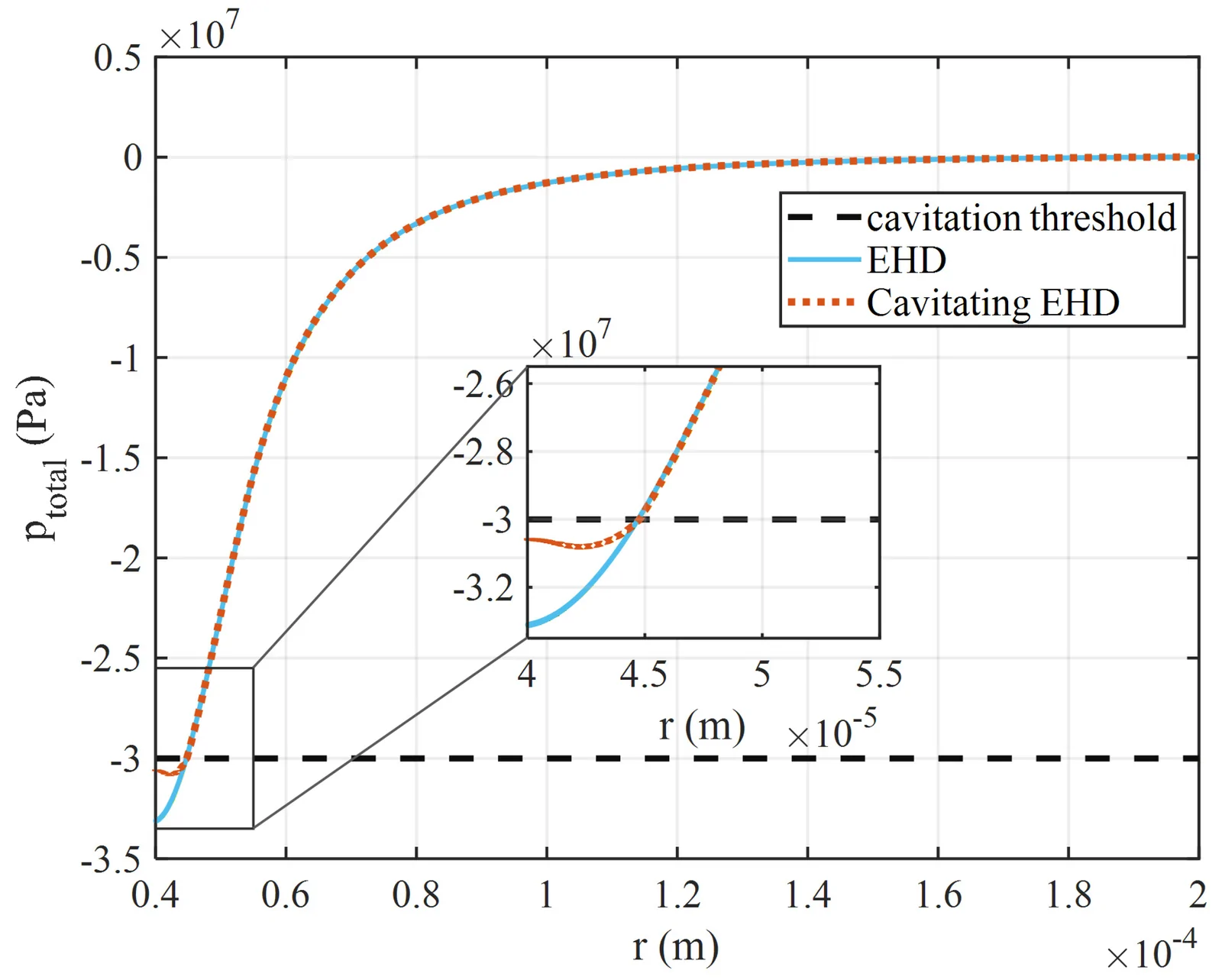
Total pressure profiles (t=10 ns) of the basic EHD model and the cavitating EHD model. The voltage rise time is set to 10 ns with a peak voltage level of 10 kV (c= 1 kV/ns).

Following the verification and validation of the basic EHD model, this work continues to incorporate the VOF into the EHD to build the cavitating EHD model. Figure 4 compares the radial distributions of total pressure from the basic EHD model discussed previously and the implemented cavitating EHD model. It is expected that in most parts of the region where the total pressures do not reach the cavitation threshold, the results from the two models are essentially the same. However, in the vicinity of the inner electrode surface, the cavitating EHD model predicts less negative pressure. The EHD model as a continuum model becomes invalid once cavities form; therefore, it can only be used to find out whether the condition is met for cavitation initiation. The cavitating EHD model, on the other hand, provides a description of cavitation dynamics once the total pressure drops below the threshold pressure. As a result of cavitation generating voids with near-zero pressure inside, the average tensile stress in the fluid–cavity mixture is thus “relieved”. The results from the cavitating EHD model are consistent with the literature [6,18] where the detailed physical mechanism of the pressure relaxation was discussed.

**Figure 5:**
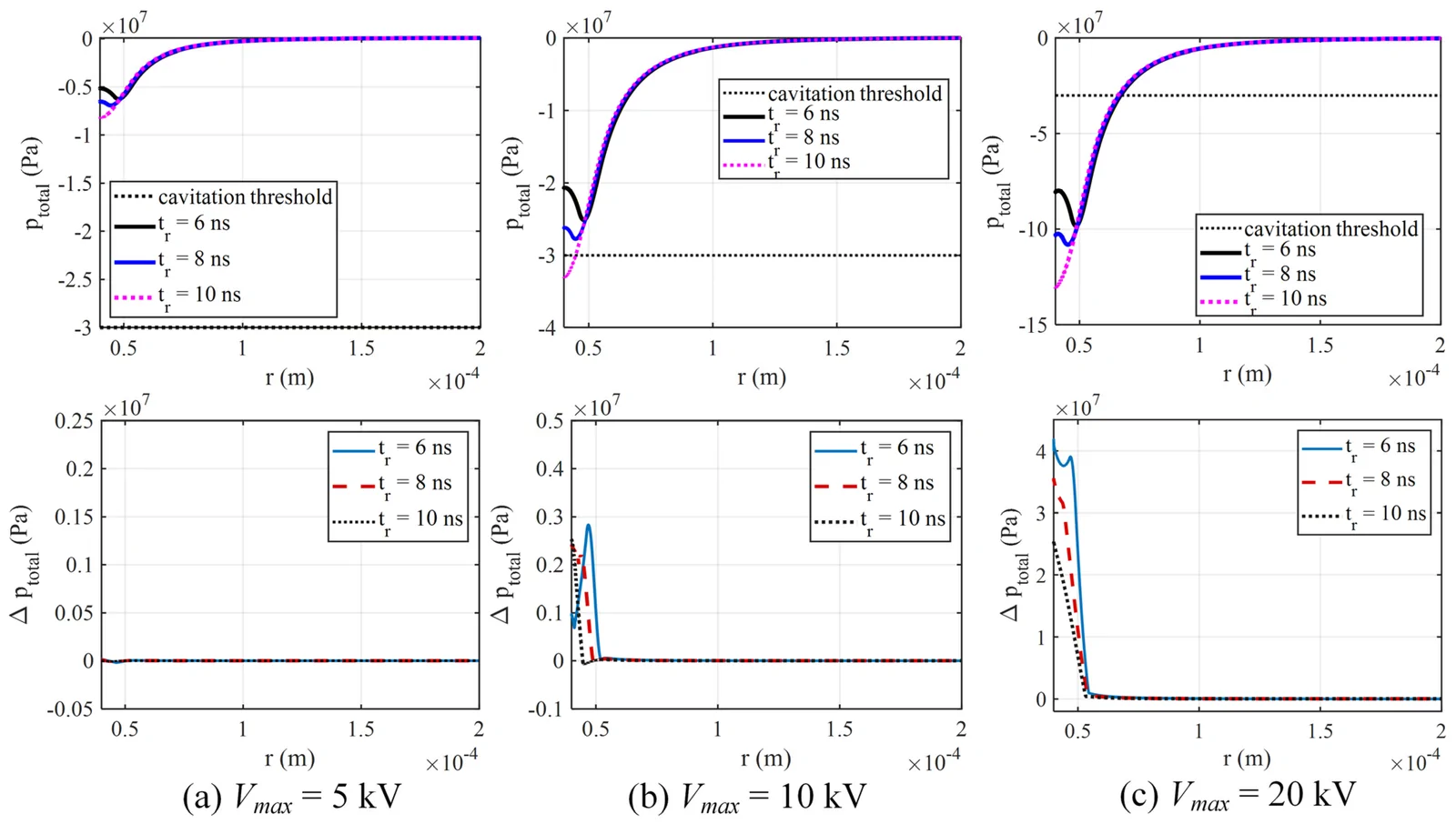
Total pressure profiles (t=10 ns) under various voltage levels and rise times using the cavitating EHD model. The maximum voltage $V_{max}$ is set to 5 kV (**a**), 10 kV (**b**), and 20 kV (**c**), with the rise time $t_{r}=$ 6, 8, and 10 ns. The three subplots at the bottom are the differences in total pressure between the cavitating EHD model and the basic EHD model.

Figure 5 expands on the behavior of the cavitating EHD model under various peak voltage levels ($V_{max}$) and rise times ($t_{r}$). By comparing with the results seen in Figure 2 and Figure 3, the profiles of total pressure under the same conditions are similar between the basic EHD model and the cavitating EHD model. In Figure 5a, when the voltage level is relatively low and no cavitation occurs, the two sets of results are the same. In Figure 5b,c, however, in regions with cavitation, the cavitating EHD model consistently predicts lower tensile stress than the basic EHD model due to the pressure relaxation. The difference increases with increasing voltage levels and can be used to indicate the location and intensity of cavitation development. In the case of $t_{r}$ = 6 ns, the region of intensive cavitation starts propagating away from the inner electrode surface once the voltage plateaus.

**Figure 6:**
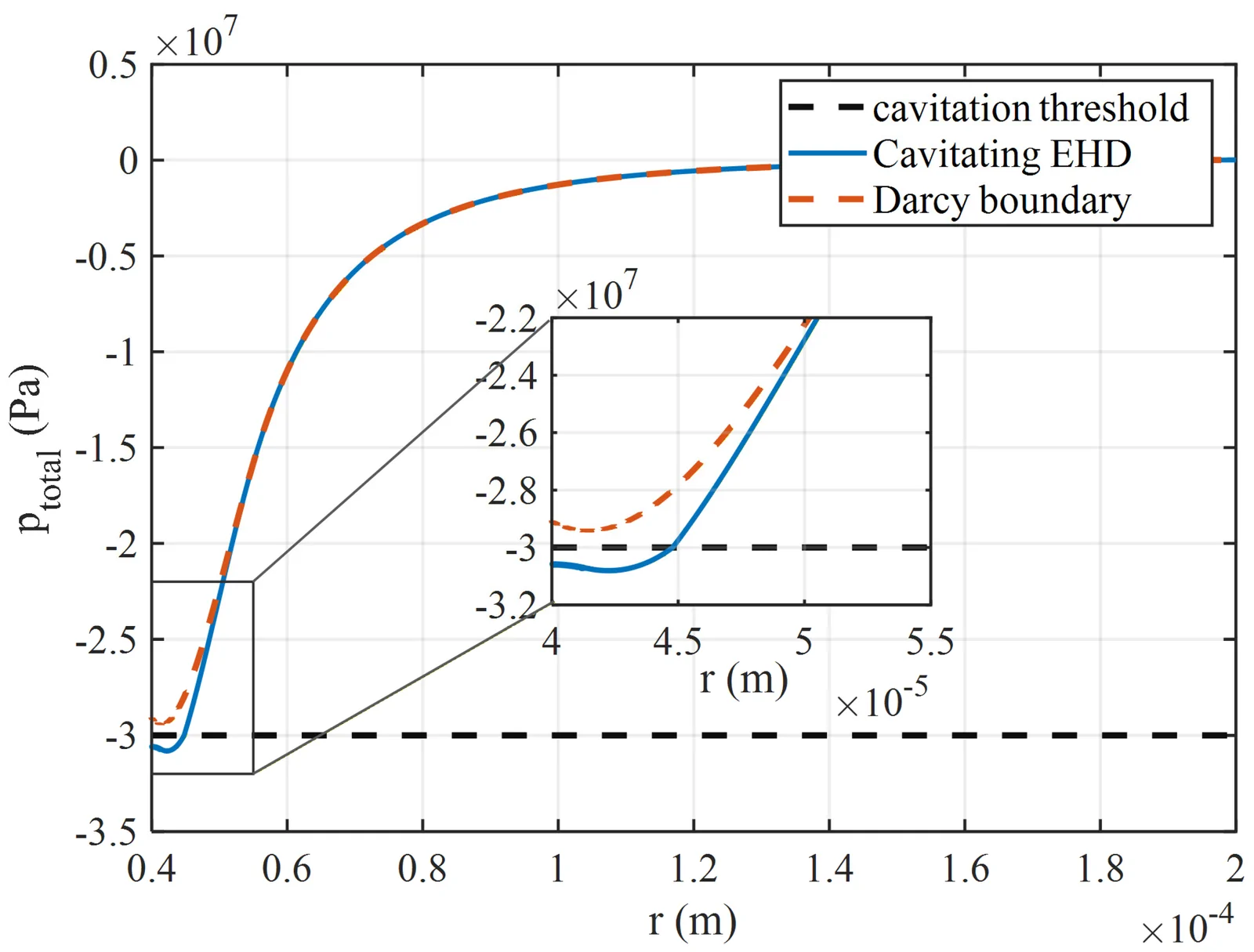
Total pressure profiles (t=10 ns) using the cavitating EHD model with and without the Darcy boundary condition. The voltage rise time is 10 ns with a peak voltage level of 10 kV. The Darcy’s law coefficient k/μL is set to $10^{-7}$ (unit: m/Pa·s).

Next, the Darcy’s boundary condition, Equation (10), is implemented in the cavitating EHD model. Figure 6 shows that, keeping other conditions the same, the Darcy boundary condition lowers the tensile stress near the inner electrode surface. This further drop in pressure is due to the water outflow into the EHD domain as a response to the large pressure difference across the permeable electrode surface. This local fluid motion is expected to relax the negative pressure buildup to a certain extent. In particular, the case presented in Figure 6 suggests that if the electrode surface provides the Darcy’s boundary condition, cavitation could be suppressed in the sense that the total pressure never reaches the cavitation threshold. This is evidence for the feasibility of the proposed conceptual design of a high-voltage electrode with a bio-inspired surface. Through the regulation of the flow field, the electrode surface could potentially affect the initiation and development of cavitation.

**Figure 7:**
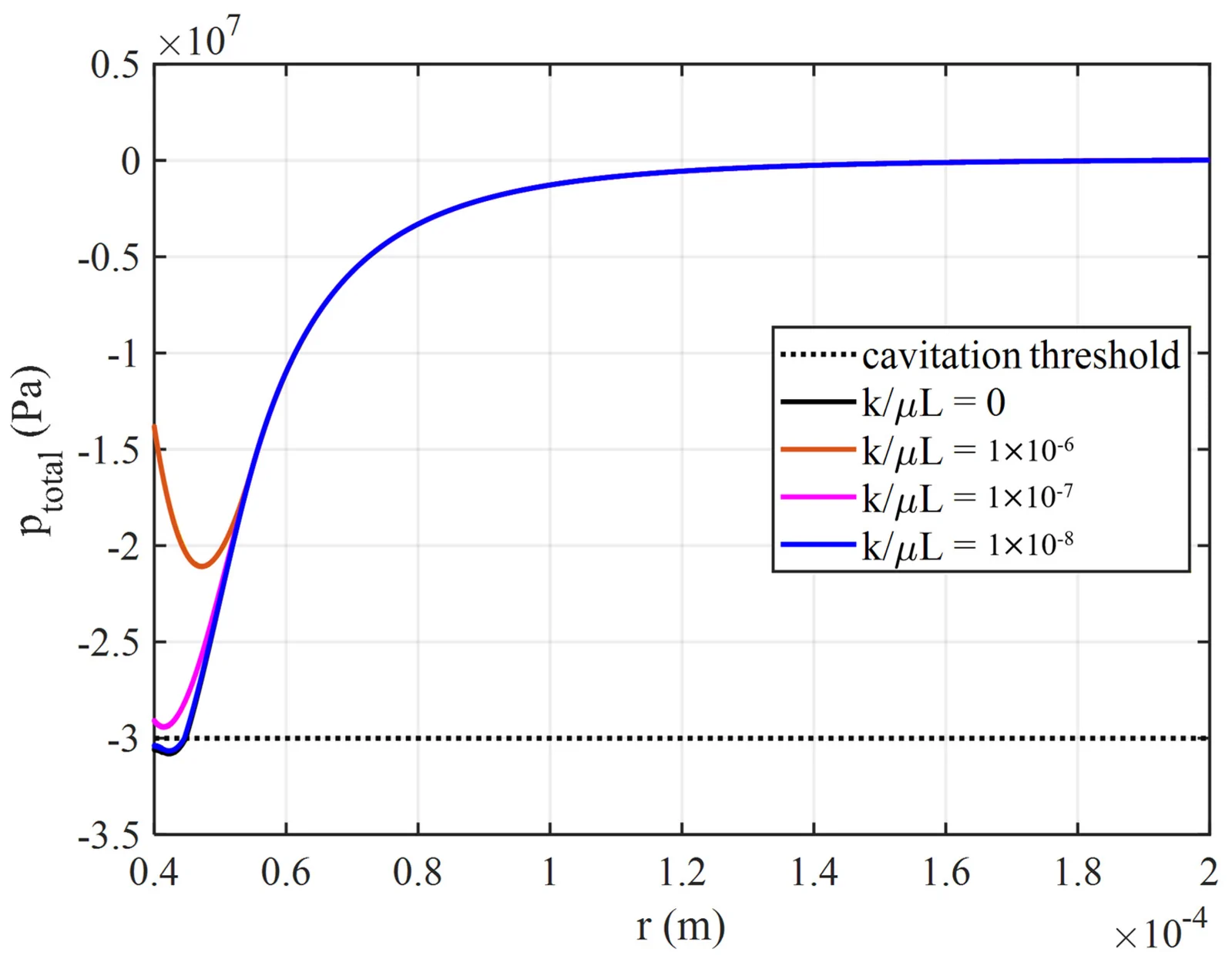
Total pressure profiles (t=10 ns) for different Darcy’s law coefficients. The voltage rise time is 10 ns with a peak voltage level of 10 kV. The Darcy’s law coefficient k/μL is set to $10^{-6}$, $10^{-7}$, and $10^{-8}$ (unit: m/Pa·s).

In Figure 7, the Darcy’s law coefficient is adjusted by an order of magnitude in both directions to test the resulting effect on the total pressure. As expected, if k/μL is very low, e.g., the permeability of the material (k) is small, no substantial differences can be observed between the results with and without the Darcy boundary condition. As the value of k/μL increases, the effect of inhibiting negative pressure buildup becomes more prominent. In this work, we have $\mu \sim 10^{-3}$ Pa·s for water, and $L\sim 10^{-5}$ m as the thickness of the electrode surface; the only variable is k, which, for known materials, could span over 15 orders of magnitude, typically from 10^−20^ to 10^−5^ m^2^. A material with $k\sim 10^{-15}$ m^2^ has a similar permeability to limestone; in this regard, the required material property is not physically unrealistic.

## 4. Discussion

A number of assumptions and simplifications have been made in the models used in this work. First, the basic EHD model assumes that the electric field is static and time-invariant, there is no space charge present, the liquid is described by the Tait equation, and the viscosity is negligible at the ns timescale. Second, the cavitation EHD model, based on the VOF method, adopts the same dynamic equations of cavitation from thermal fluid systems with much larger timescales and only considers homogeneous cavitation. Third, the simplest form of the Darcy boundary condition is used, while there are nonlinear and time-dependent versions. Subsequent work can be conducted to extend and further validate this study in the following directions: (a) including the effect of cavitation on the electric field distribution, i.e., the presence of cavities lowers the local electric permittivity and affects the local electric field; (b) exploring the applicable limits of the cavitation dynamic equations, especially when investigating the first ns of electrostrictive cavitation; (c) studying more sophisticated forms of Darcy’s law to generate more realistic flow patterns and pressure distributions.

This work presents a self-consistent computational study of the conceptual design of a bio-inspired high-voltage electrode surface. Although it will be the task of future works to fabricate the proposed electrode and test whether it could inhibit or delay cavitation inception, a few comments are to be made here regarding the implementation and limitations of the proposed design. First, the electrode material should be a good electrical conductor and with sufficient mechanical strength (it could be rigid or compliant, but not prone to fracturing). The most important parameters are permeability and thickness, as indicated in the Darcy’s law equation (Equation (10)). The range of values of permeability for the materials to be used is found to be reasonable in this work. Second, the method used for electrode surface fabrication and treatment needs to ensure that the surface roughness is appropriate for a high-voltage electrode. It is well-known that large length-to-diameter-ratio protrusions on an otherwise smooth electrode surface would intensify the local electric field. For the permeable electrode surface, if the pore size is below 1 micron, the overall roughness is similar to that of a very smooth metal electrode surface. Finally, the proposed electrode concept might have some limitations or disadvantages in real-world applications. For instance, the durability of the permeable material could be an issue, especially when it is subject to repeated electrical discharges.

## 5. Conclusions

In this work, the continuum EHD model is coupled with a cavitating mixture model to shed new light on electrostrictive cavitation, which is the initial stage of electrical breakdown in dielectric liquids under ns/sub-ns pulsed high voltages. It has been demonstrated that the cavitating EHD model is a novel approach with which to gain insights into the characteristics and dynamics of the cavitation zone. Cavitation development results in a reduction in the local tensile stress (negative pressure). Applying the Darcy boundary condition at the high-voltage electrode, the tensile stress near the electrode surface is further reduced.

Inspired by cell membranes, an innovative electrode design concept is proposed, in which the electrode is hollow with a permeable enclosure, allowing liquid flow in response to a pressure difference between the interior and the environment. The numerical modeling results support the idea that the bio-inspired electrode surface holds potential to suppress cavitation and subsequent breakdown.

## Data Availability

The data that support the findings of this study are available from the authors upon reasonable request.
